# Psychometrics of the self-efficacy for physical activity scale among a Latina women sample

**DOI:** 10.1186/s12889-018-5998-0

**Published:** 2018-09-05

**Authors:** Andrea S. Mendoza-Vasconez, Becky Marquez, Tanya J. Benitez, Bess H. Marcus

**Affiliations:** 1 Department of Family Medicine & Public Health, University of California San Diego, 9500 Gilman Drive, San Diego, CA 92093-0725 USA; 2 Graduate School of Public Health, San Diego State University, 5500 Campanile Dr, San Diego, CA 92182 USA; 3 Department of Behavioral and Social Sciences, Brown University School of Public Health, 121 South Main Street, Providence, RI 02903 USA

**Keywords:** Hispanic, Validity, Reliability, Factor analysis, Exercise, Health, IRT

## Abstract

**Background:**

Even though Latinos have become a priority population for the promotion of physical activity in the United States, several widely used scales in physical activity promotion research have not been validated among this population, particularly in Spanish. This study aims to assess the validity and other psychometrics of the Self-Efficacy for Physical Activity scale among a sample of Spanish-speaking Latina women who participated in the Pasos Hacia La Salud intervention. We also explored alternatives for scale simplification.

**Methods:**

Data from 205 women corresponding to baseline, 6-month, and 12-month time points were analyzed. Internal consistency was assessed. A series of Spearman correlations, t-tests, linear regressions, and logistic regressions were used to assess the concurrent and predictive validity of the Self Efficacy for Physical Activity scale against both self-report and accelerometer-measured physical activity, using both continuous and categorical outcome data. Item Response Theory and factor analysis methods were used to explore alternatives to simplify the scale. Psychometric tests were repeated with the simplified scale.

**Results:**

Cronbach’s alpha for the original scale was .72, .76, and .78 for baseline, 6-month, and 12-month data respectively. All concurrent validity tests conducted with 6-month and 12-month data, but not with baseline data, were statistically significant. Self-efficacy at 6 months was also predictive of physical activity at 12 months for all tests except one. Based on plots of Option Characteristic Curves, a modified version of the scale was created. Psychometric results of the modified scale were similar to those of the original scale.

**Conclusions:**

This study confirmed the scale’s reliability and validity, and revealed that the scale’s accuracy improves when some response items are collapsed, which is an important finding for future research among populations with low literacy levels.

## Background

Physical activity (PA) promotion among Latinos in the United States has drawn much attention from the scientific community in recent years for multiple reasons. Mainly, Latinos are the largest ethnic minority in the US; according to national data from the US Census Bureau, 17.3% of the population in the US self-identifies as Hispanic or Latino, an ethnic category that includes individuals of different races, nationalities, and immigrant generations [1]. Additionally, Latinos suffer disproportionately from many chronic illnesses [2, 3] that can be prevented through modification of lifestyle factors, including engagement in PA. Despite the health enhancing benefits of PA, Latinos are less likely than non-Hispanic White populations to report meeting 2008 National PA guidelines for moderate to vigorous PA [4], which might heighten their risk of developing related chronic illnesses. Among Latinos, women in particular are less likely to be sufficiently active compared to men [5, 6]; for example, in a study of different Latino subgroups, between 15 and 29% of Latina women of different subgroups reported meeting PA recommendations, compared to 25% to 35% of Latino men [5]. Additionally, Latina women are at higher risk of various diseases related to lack of PA, such as diabetes, compared to Latino men and non-Latina women [7]. As a result, in the last decade there has been an increased interest in PA promotion among Latina women in the US, and various theory-based PA interventions have been developed or culturally adapted to meet the needs of this at-risk population [8].

The use of theory-based interventions is essential for reducing lifestyle-related chronic illness and health disparities as it helps to understand the dynamics of behavioral change, and provides a framework to guide the development and evaluation of appropriate interventions [9]. Social Cognitive Theory (SCT) is one of the most commonly used theories in the promotion of PA among this group [8]. SCT posits a reciprocal relationship between personal and environmental factors; in other words, behavior influences and is influenced by personal factors and by environmental factors [10]. Self-efficacy, which refers to a person’s belief (confidence) in his or her ability to engage in a behavior [10], is a key construct of SCT. Self-efficacy has been consistently found to predict behavior across different populations and health behaviors [11]; it has been recognized as an important construct in the study of behavior change, and has thus been incorporated as a construct in many other behavioral theories, such as the Transtheoretical Model (TTM), the Health Belief Model, and the Theory of Planned Behavior [12]. The ability to accurately measure self-efficacy across different populations and behaviors is important for the advancement of health behavior research.

The Self-Efficacy for Physical Activity (SEPA) scale is a 5-item measure developed by Marcus and colleagues [13], which has been used in many PA promotion studies among both Latino and non-Latino populations [14–17]. The scale assesses an individual’s confidence for engaging in exercise in the presence of barriers (Table 1). Previous studies have confirmed the scale’s reliability and validity among different populations, such as male and female workers in Rhode Island and in Australia, and Finnish and American college students [18–20]. To our knowledge, the psychometrics of the scale have not been assessed among samples of Latina women.

**Table 1:** Self-Efficacy for Physical Activity Scale

English						Spanish					
*I am confident I can participate in regular exercise when:*	Not Confident	Slightly Confident	Moderately Confident	Very Confident	Extremely Confident	*Cuan confiada esta usted en que pueda hacer ejercicio en las siguientes situaciones:*	No confiada	Poco confiada	Moderadamente confiada	Muy confiada	Extremadamente confiada
1. I am tired.	1	2	3	4	5	1. Cuando estoy cansada.	1	2	3	4	5
2. I am in a bad mood.	1	2	3	4	5	2. Cuando estoy de mal humor.	1	2	3	4	5
3. I feel I don’t have time.	1	2	3	4	5	3. Cuando siento que no tengo tiempo.	1	2	3	4	5
4. I am on vacation.	1	2	3	4	5	4. Cuando estoy de vacaciones.	1	2	3	4	5
5. It is raining or snowing.	1	2	3	4	5	5. Cuando esta lloviendo o nevando.	1	2	3	4	5

Because the SEPA scale has been adapted for Spanish language, and cultural and gender factors might influence participants’ responses, there is a need to evaluate the psychometrics of this increasingly utilized measure among Latina women. Additionally, although self-efficacy is one of the most common psychosocial constructs studied in relation to PA, in Latina women results are mixed, leading some researchers to question the validity of this individual-level construct for a collectivist oriented group [21]. Some of the disagreement may stem from the diversity in study design, self-efficacy measure, PA measure, and type of PA assessed. The vast majority of studies are cross-sectional [22–29], based on self-reported PA [22–29], focus on leisure time PA [23, 28], or have a dichotomous PA outcome [24–29]. Among PA interventions for Latinos, changes in self-efficacy are often reported, yet the relationship between self-efficacy and changes in PA is rarely reported [17, 21, 30–32]. Such data are important for identifying mechanistic pathways to PA adoption and maintenance.

The main objective of this study was to assess the reliability, concurrent validity, and predictive validity of the SEPA scale among a sample of adult Latina women participating in Pasos Hacia la Salud, a 12-month culturally and linguistically adapted Internet-based PA intervention [32, 33]. Additionally, given the paucity of studies assessing the validity of this and other self-efficacy scales against objective measures of PA, this study used both self-reported and accelerometer-measured moderate to vigorous physical activity (MVPA) for validation purposes. As a secondary objective, this study explored different alternatives to simplify the SEPA scale, applying techniques such as exploratory factor analysis [34] to evaluate the contribution of each scale item to the assessment of self-efficacy, and Item Response Theory (IRT) methods [35] to explore the possibility of collapsing response options for each item.

## Methods

### The Pasos Hacia La Salud intervention

Pasos Hacia la Salud [32, 36] was a 12-month randomized controlled trial of a culturally and linguistically adapted, individually-tailored, Internet-based intervention to promote PA in Latina adults. Participants were inactive Latina women between the ages of 18–65 years, randomized to either a Spanish language Internet-based PA Intervention Group or a Spanish language Wellness Contact Control Internet Group. The PA Intervention was based on the Social Cognitive Theory [10] and the Transtheoretical Model [37] and emphasized theoretically-driven strategies for increasing PA (e.g., self-monitoring, goal setting, increasing social support). Pasos Hacia la Salud was conducted at the University of California San Diego. All study procedures were approved by the University’s Institutional Review Board and participants provided written informed consent. Data for this study were collected between December 2011 and September 2014.

### Study participants

Adult Latina women (*N* = 205) enrolled in the study provided baseline, 6- and 12-month data regarding their PA, their self-efficacy for PA, and their use of cognitive and behavioral strategies for PA behavior change. All data collection was conducted using Spanish versions of each questionnaire. More details about the study and data collection procedures are described elsewhere [36]. Briefly, participants were recruited through multiple methods including Craigslist and local newspaper ads, flyers posted and distributed at local stores, churches, health-related community events, mailings through primary care doctor offices, and other participant referrals. To be eligible for the intervention, participants had to be self-identified Hispanic/Latino women between the ages of 18 and 65, report engaging in less than 60 min per week of MVPA, and meet certain health requirements, including BMI < 45 kg/m2, and not having a history of coronary heart disease, diabetes, stroke, or any serious medical conditions that would render PA unsafe. Additionally, participants had to be able to read and speak Spanish fluently, have no plans to become pregnant or move away from the area during the study period, have access to Internet, score at least adequate in the Short Test of Functional Health Literacy in Adults (STOFHLA), and be willing to be randomized to an Internet based PA intervention or a wellness control condition.

### Measures

#### Self-efficacy for physical activity (SEPA) scale

The scale, consists of five items assessing confidence in participating in exercise in the presence of barriers such as feeling tired, being in a bad mood, not having time, on vacation, and experiencing bad weather (Table 1). Response options are on a five point Likert-scale, ranging from not confident to extremely confident. Research suggests this scale is one-dimensional [19], meaning that all the scale’s questions are part of a unique construct, according to factor analysis results. The scale has also shown acceptable reliability and validity among different populations [13, 18, 20].

The following measures were used for validation purposes in this analysis:

#### Seven-day physical activity recall (7-day PAR)

The 7-Day PAR is an interviewer-administered self-report measure that inquires about MVPA over the past week in at least ten-minute bouts across various contexts (leisure, occupational, transport etc.). It has shown good reliability, validity, and sensitivity to change over time [38], in Latino and non-Hispanic White populations [39].

#### Accelerometry

Participants wore the ActiGraph GT3X+ accelerometer on their left hip during the seven days leading up to their self-report measurement visit. Data were processed using the ActiLife software, 60 s epochs, and with a cut point of 1952 for moderate PA [40]. Per standard procedures used in the field, while participants were asked to wear the accelerometer for a minimum of 12 h per day, they were only asked to re-wear the accelerometer if they did not meet a minimum requirement of 5 days of at least 600 min of wear time. Accelerometers are the gold standard in the measurement of PA, and have been validated with heart rate telemetry and total energy expenditure [40, 41]. Minutes of MVPA were added up to create a continuous variable (only bouts of at least ten minutes were considered), used for concurrent and predictive validation of the SEPA scale.

The 7-Day PAR and accelerometer measure different aspects of PA. Unlike the 7-Day PAR, accelerometers do not accurately estimate activities such as stationary bicycling, elliptical training, swimming, and upper extremity movement. Both 7-day PAR and accelerometer-measured MVPA are reported in minutes per week of MVPA. Additionally, two dichotomous variables were created (one with 7-day PAR data and one with accelerometer data) to distinguish individuals who were meeting 2008 National PA guidelines [4] and those who were not. These variables were created by adding bouts of at least 10 min of MVPA; those who had at least 150 min of MVPA per week were categorized as meeting guidelines.

### Data analysis

All analyses were conducted using RStudio Version 0.99.486. Descriptive statistics were used to analyze the demographic data of the sample, collected at baseline. Additionally, frequencies and percentages, or means and standard deviations, were obtained for the variables of interest for this analysis. The following analyses were conducted using baseline, 6-month, and 12-month data from the SEPA scale.

#### Internal consistency

Cronbach’s alpha was calculated to assess the internal consistency of the SEPA scale.

#### Concurrent validity

Using Spearman correlations, we assessed the correlation between SEPA scores and PA (measured with the 7-day PAR and the accelerometer) at baseline, 6 months, and 12 months. Additionally, those who were meeting and not meeting PA guidelines at 6 and 12 months were compared regarding their self-efficacy using a series of independent samples *t*-tests; baseline data were not analyzed using *t*-tests because it was restricted by design so that no participants would meet PA guidelines.

#### Predictive validity

To determine whether self-efficacy at baseline could predict PA at 6 months, and whether self-efficacy at 6 months could predict PA at 12 months, we created a series of linear regression models. For these models, PA (measured with the 7-day PAR and the accelerometer) was the outcome variable, while self-efficacy was the predictor variable; we also controlled for baseline self-efficacy and PA. We assessed all model assumptions and used square-root transformation for the dependent variable to meet the assumption of normally distributed residuals and homoscedasticity.

To accomplish the study’s secondary objective to assess different alternatives to compress the SEPA scale, the following analyses were conducted using baseline, 6-, and 12-month data from the SEPA scale:

#### Non-parametric kernel smoothing techniques and option characteristic curves

Item response theory (IRT) methods [35] were then used to model the association between each item in the SEPA scale, and the latent trait of self-efficacy for PA. IRT is a theory of testing that has been mainly used in the field of education (and is increasingly being used in health fields) to create assessment tools that adapt to individuals’ level or severity of a trait [35]. Non-parametric Kernel Smoothing techniques developed by Ramsay [42] were used to plot Option Characteristic Curves (OCC). OCC plots indicate the probability of selecting a particular response for each question, in the context of the person’s overall self-efficacy level. These plots were used to explore the possibility of collapsing response options for each item if curves overlapped (i.e. if response options did not discriminate enough between individuals with different levels of self-efficacy).

#### Exploratory factor analysis

Exploratory factor analysis techniques [34] were used to assess the unidimensionality of the SEPA scale and the contribution of each scale item to the assessment of self-efficacy.

Similar internal consistency, concurrent validity, and predictive validity tests were conducted with the SEPA scale that had collapsed response options to determine whether reliability and validity improved, decreased, or were sustained.

## Results

### Descriptive analysis

Results of descriptive statistics are presented in Table 2. Briefly, most participants (83%) reported being of Mexican origin or descent, and most (86%) completed high school. Additionally, 46% of participants were not employed and 40% reported an annual household income less than US $20,000. On average, participants at baseline were engaging in 32 min of MVPA per week, measured with the accelerometer. At 6 and 12 months, participants were engaging in 59 and 62 min of MVPA per week on average, respectively. For self-report MVPA, participants reported engaging in 9, 87, and 92 min of MVPA per week at baseline, 6 months, and 12 months, respectively. Mean self-efficacy at baseline was 11.5 (on a scale of 5 to 25), while at 6 months it was 12.4 and at 12 months 12.9.

**Table 2:** Sample characteristics (*N* = 205)

Categorical variable	N (%)
Educational level	
Less than 12 years (did not complete High School)	29 (14.2)
Graduated from High School	97 (47.5)
Graduated from College	78 (38.27)
Ethnicity (sub-group)	
Mexican	173 (83.4)
Other	32 (15.6)
Work status	
Not employed	92 (45.3)
Full-time	55 (27.1)
Part-time	56 (27.6)
Annual Income (US $)	
Less than $19,999	83 (40.5)
$20,000 to $39,999	80 (39.1)
$40,000 or more	33 (16.1)
Unknown	9 (4.4)
Continuous variable	Mean (SD)
Age	39.2 (10.5)
Self-Efficacy (Original scale, Baseline)	11.7 (4.0)
Self-Efficacy (Original scale, 6-Months)	12.4 (4.4)
Self-Efficacy (Original scale, 12-Months)	12.9 (4.6)
Physical Activity (Accelerometer, baseline)	32.3 (60.0)
Physical Activity (Accelerometer, 6-Months)	59.2 (78.7)
Physical Activity (Accelerometer, 12-Months)	62.4 (80.4)
Physical Activity (PAR, baseline)	9.2 (19.9)
Physical Activity (PAR, 6-Months)	86.7 (95.6)
Physical Activity (PAR, 12-Months)	91.9 (99.8)

While baseline data for 205 women was available, only 142 and 150 of those women provided all the 6- and 12-month information, respectively, used for this secondary data analysis. Upon closer inspection, we found no significant differences in baseline data for demographic and other variables, including no differences in self-efficacy or PA, between those who provided all 6 and 12-month data and those who did not. The only exception was the variable age: mean age for responders was 41.2 years, while for non-responders it was 35.1 years (*t* = − 3.67, df = 160).

### Internal consistency

Cronbach’s alpha was .72 (95% CI .62–.82) for the baseline data, .76 (95% CI .67–.85) for the 6-month data, and .78 (95% CI .69–.87) for 12-month data, indicating acceptable internal consistency.

### Concurrent validity

There were positive associations at 6 and 12 months between SEPA scores and minutes/week of MVPA (measured with the 7-day PAR and the accelerometer). As shown in Table 3, the correlation coefficients for 6- and 12-month data were all statistically significant at the 0.05 level, and ranged from small to medium. As expected due to the narrow range of baseline SEPA and MVPA values, which were restricted by design for the purposes of this intervention, we did not observe associations between baseline PA and baseline self-efficacy.

**Table 3:** Concurrent and Predictive Validity Tests for Original SEPA Scale

	SEPA at 6 months	SEPA at 12 months
7-day PAR at 6 months (minutes/week)	ρ = 0.464**	n/a
Accelerometer 6 months (minutes/week)	ρ = 0.273**	n/a
7-day PAR 6 months (meeting guidelines)	*t*(40) = −5.605**	n/a
Accelerometer 6 months (meeting guidelines)	*t*(18) = − 2.789*	n/a
7-day PAR at 12 months (minutes/week)^a^	*B* = 0.368** SE = 0.109	ρ = 0.492**
Accelerometer 12 months (minutes/week)^a^	*B* = 0.313** SE = 0.102	ρ = 0.340**
7-day PAR 12 months (meeting guidelines)	*B* = 0.129** SE = 0.472	*t*(64) = − 5.157**
Accelerometer 12 months (meeting guidelines)	*B* = 0.081 SE = 0.058	*t*(29) = − 2.565*

**p value < .01, *p value < .05, +*p* value < .1

Note a: In linear regression models, square root transformations were used for the outcome variables (7-day PAR and accelerometer-measured MVPA)

Regarding the difference in self-efficacy scores between those who were meeting PA guidelines at 6 and 12 months, compared to those who were not, we also found significant differences. As shown in Table 3, results of independent samples t-tests (using data collected with both the 7-day PAR and the accelerometer) showed that those who were meeting guidelines had significantly higher mean self-efficacy scores compared to those who were not. For example, at 6 months, using the original self-efficacy scale, those who were meeting the guidelines (as measured by accelerometer) scored on average 15.5 (sd = 4.872) out of 25 in self-efficacy, compared to 11.95 (sd = 4.271) among those who were not meeting guidelines.

### Predictive validity

Multiple linear regression models revealed that SEPA scores at 6 months significantly predicted MVPA at 12 months (self-report and accelerometer-measured). As shown in Table 3, greater self-efficacy was associated with more minutes of MVPA per week. Additionally, self-efficacy at 6 months predicted meeting PA guidelines at 12 months when self-report PA data were used, but not with accelerometer data, so that for every unit increase in self-efficacy, we would expect to see a 14% increase in the odds of meeting PA guidelines. As expected because of the narrow range of the SEPA baseline values, baseline SEPA scores did not predict MVPA engagement at 6 months, nor meeting PA guidelines at 6 months.

### Non-parametric kernel smoothing techniques and option characteristic curves

**Fig. 1:**
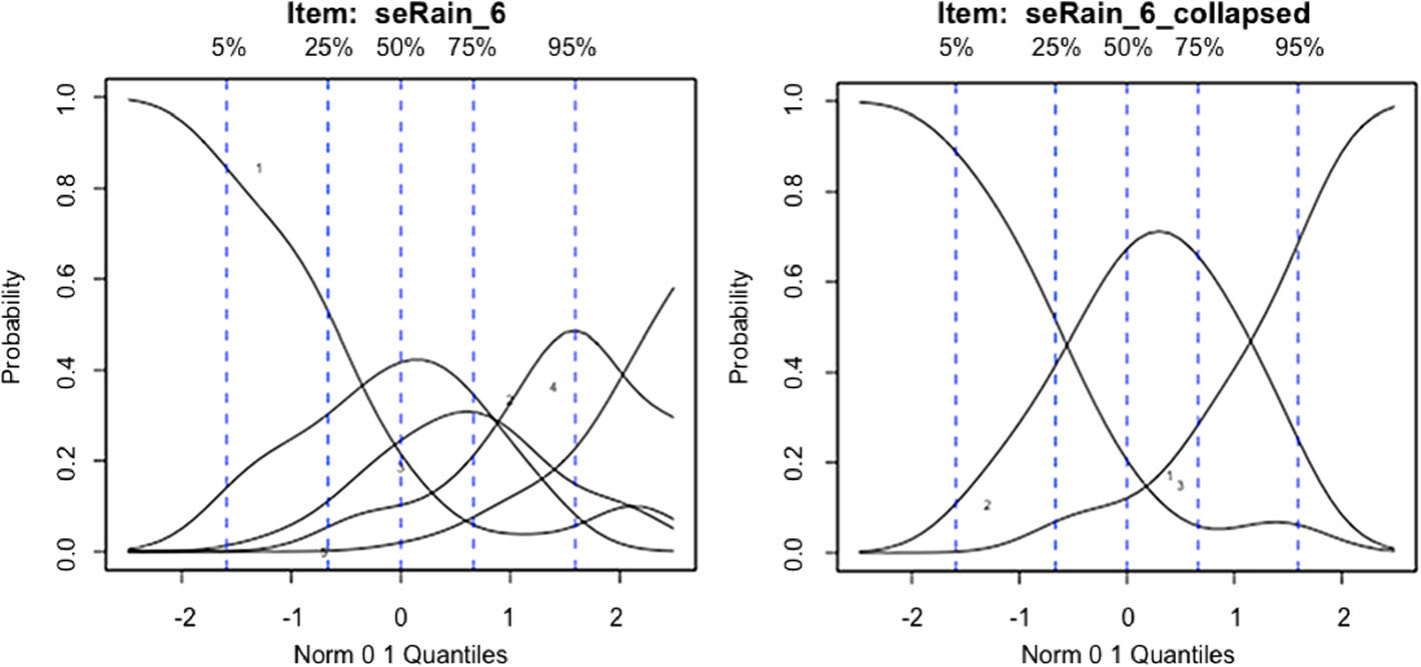
Example of original and collapsed OCC plots. Original and collapsed OCC plots for the fifth item (pertaining exercising when it is raining) of the Self-Efficacy for Physical Activity Scale, using 6-month data

Using Item Response Theory [35], specifically non-parametric Kernel Smoothing techniques [42], we plotted Option Characteristic Curves (OCC) for each of the items in the SEPA scale. These curves represent an individual’s probability of choosing one of the five response options, based on his or her overall severity in the latent self-efficacy construct. Based on OCC plots, which showed overlapping probabilities for response options, we collapsed each of the item’s five response options into three for increased accuracy: (1) Not Confident, (2) Slightly Confident/Moderately Confident, and (3) Very Confident/Extremely Confident. Figure 1 contains an example of the plots of non-parametric OCCs for question #5 (pertaining to exercising when it is raining) for 6-month data.

### Exploratory factor analysis

For exploratory factor analysis, using a scree plot and parallel analysis [43], we decided to keep one factor for both the original scales and the scales with collapsed response options, for baseline, 6-month, and 12-month data. For the original scale, factor loadings ranged from .54 to .72 with baseline data. As shown in Table 4, the vacation item had the lowest factor loading (.54), with a shared variance of .29, and a unique variance of .71. Similar results were observed for 6 and 12-month data, as depicted in Table 4. Additionally, similar results were observed for the scale with modified response options, as depicted in Table 4. Consistently, the vacation item had the lowest factor loadings, the lowest shared variance, and the highest unique variance.

**Table 4:** Internal Consistency and Factor Analysis Tests with Baseline, 6-month, and 12-month Data

	Original Scale (Baseline)		Collapsed Scale (Baseline)	
	All Five Items	Vacation Dropped	All Five Items	Vacation Dropped
Cronbach’s Alpha	.72 (95% CI .62–.82)	.70 (95% CI .58–.82)	.71 (95% CI .61–.81)	.67 (95% CI .54–.79)
Highest Factor Loading: Item 1 (Tired)	.72	.80	.68	.77
Lowest Factor Loading: Item 4 (Vacation)	.54	–	.63	–
	Original Scale (6-months)		Collapsed Scale (6-months)	
	All Five Items	Vacation Dropped	All Five Items	Vacation Dropped
Cronbach’s Alpha	.76 (95% CI .67–.85)	.78 (95% CI .68–.88)	.76 (95% CI .67–.85)	.75 (95% CI .64–.86)
Highest Factor Loading: Item 1 (Tired)	.86	.94	.81	.87
Lowest Factor Loading: Item 4 (Vacation)	.4	–	.54	–
	Original Scale (12-months)		Collapsed Scale (12-months)	
	All Five Items	Vacation Dropped	All Five Items	Vacation Dropped
Cronbach’s Alpha	.78 (95% CI .69–.87)	.80 (95% CI .70–.90)	.78 (95% CI .69–.87)	.79 (95% CI .69–.89)
Highest Factor Loading: Item 1 (Tired)	.89	.95	.92	.97
Lowest Factor Loading: Item 4 (Vacation)	.42	–	.42	–

### Internal consistency of the modified scales

Based on the results of OCC plots and exploratory factor analysis, we explored the effects of dropping the vacation item and collapsing the response options on the scale’s internal consistency. Table 4 summarizes the results of the internal consistency and factor analysis tests with baseline, 6-, and 12-month data. We were unable to detect significant differences in internal consistency between the original and the modified scales, as revealed by the overlapping confidence intervals.

Given the results of factor analysis and internal consistency tests, we decided to continue with a five-item scale. We opted to maintain the 5-item scale given the history of its use among different populations. Nevertheless, we decided to collapse the five response options into three for subsequent analyses, as this has the potential to decrease participant burden, and it improved the precision of the scale, increasing the amount of shared variance for items in factor analyses, and reducing the amount of unique variance.

### Concurrent and predictive validity of the modified scale

As shown in Table 5, results of concurrent and predictive validity of the modified scale with collapsed response options were very similar to the results for the original scale.

**Table 5:** Concurrent and Predictive Validity Tests for SEPA Scale With Collapsed Response Options

	SEPA at 6 months	SEPA at 12 months
7-day PAR at 6 months (minutes/week)	ρ = 0.459**	n/a
Accelerometer 6 months (minutes/week)	ρ = 0.295**	n/a
7-day PAR 6 months (meeting guidelines)	*t*(43) = −5.945**	n/a
Accelerometer 6 months (meeting guidelines)	*t*(18) = −2.979**	n/a
7-day PAR at 12 months (minutes/week)^a^	B = 0.643** SE = 0.186	ρ = 0.429**
Accelerometer 12 months (minutes/week)^a^	B = 0.534** SE = 0.174	ρ = 0.331**
7-day PAR 12 months (meeting guidelines)	B = 0.229** SE = 0.083	*t*(71) = −4.227**
Accelerometer 12 months (meeting guidelines)	B = 0.174^+^ SE = 0.103	*t*(30) = − 2.703*

**p value < .01, *p value < .05, +*p* value < .1

Note a: In linear regression models, square root transformations were used for the outcome variables (7-day PAR and accelerometer-measured MVPA)

## Discussion

Given the increasingly frequent use of the SCT as a framework for PA promotion among Latinos [8] it is important and necessary to have validated measures of psychosocial constructs to assess changes among this population. This study aimed to assess the reliability and validity of the SEPA scale among a sample of Latina women. As found in other populations [13, 18–20], the scale showed unidimensionality (i.e. all items load on the same factor in factor analysis), acceptable internal consistency and concurrent and predictive validity.

Moreover, a modified and simplified version of the self-efficacy scale, with response options collapsed into three categories, also showed unidimensionality, acceptable internal consistency, and concurrent and predictive validity. These results apply specifically to the analysis of data collected with the SEPA scale, rather than the actual data collection. Nevertheless, they warrant future research to explore the feasibility and validity of using simplified scales with smaller number of response options in order to decrease participant burden in the data collection process.

While the five-item SEPA scale is not particularly onerous, it may be beneficial to explore ways to simplify data collection instruments in order to minimize participant burden; this is particularly important for conducting research with populations that may have lower literacy levels [44]. The advent of technology for health promotion and data collection provides avenues for such endeavours. For example, the use of IRT methods in the field of health promotion research is rapidly expanding, although still at its infancy. Baranowski and colleagues [45], for example, used IRT methods to develop a shorter scale to measure self-efficacy for the consumption of fruits and vegetables, intending to minimize participant burden. With the surge of technology-based health behavior interventions, such as the Pasos Hacia La Salud intervention, there is also an unprecedented possibility to incorporate IRT methods for measurement, which may lead to increased precision along with decreased participant burden.

For the purposes of exploring scale simplification, we also considered the possibility of dropping certain scale items with lower factor loadings. The scale item pertaining to engagement in exercise during vacation time consistently showed greater unique variance compared to other items, across different analyses. A previous study also found this item (and the item pertaining to confidence in exercising when the weather is bad) to be less correlated with the rest of the items in the scale [19]. Perhaps other items pertain more to situations that are under the individual’s control, such as mood, tiredness, and time management, while these two items pertain to more external conditions. It is also possible that Latina women may have additional responsibilities to attend to during their vacation times, such as going to visit family abroad and having to care for aging or ill parents/family members during this time. Nevertheless, for the purposes of comparability, and given the acceptable factor loadings, the acceptable amount of shared variance, and the history of the scale, it is recommended that the full five-item scale be used in future studies.

This study is not without limitations, particularly given its reliance on secondary data for analyses. Our sample consisted predominately of Latina women of Mexican origin or descent who were recruited using non-probability sampling techniques. Thus, these results are not generalizable to the larger Latino population living in the US, particularly because the SEPA scale assesses self-efficacy in the context of barriers, and these may or may not similarly apply to women of other Latino subgroups or Latino men. Moreover, the data used in this study were obtained from an interventional study, where people who were engaging in PA at baseline were excluded from the study. Thus, certain measures used in this analysis have narrow ranges, especially at baseline. However, it may be possible to consider some of these narrow ranges as further evidence of measure validity; for example, the narrow range in the PA variables at baseline should translate to a narrow range in self-efficacy for PA. Additionally, the long time that elapsed between measures may have weakened our predictive validity results. Despite these weaknesses, the use of longitudinal data from an intervention study may also be considered a strength, as the majority of existing validation studies have used cross-sectional designs. Nevertheless, future studies may consider using longitudinal data that have closer time points and wider ranges, which are not restricted by the context of an intervention. Another limitation pertains the construction of the dichotomous variable for *Meeting PA Guidelines,* which was based on the cut point of 150 min of MVPA without a distinction between moderate and vigorous PA. Nevertheless, this approach to dichotomizing the *Meeting PA Guidelines* variable has been extensively used in the literature previously [46–48], particularly among samples of participants who engage in mostly moderate PA and very little vigorous PA, as is the case with our sample.

There are several additional strengths to this study. We confirmed the reliability, concurrent and predictive validity, and the unidimensionality of the SEPA scale among this sample of Latina women. We also assessed validity against PA as self-reported and objectively measured, as well as continuously (i.e. minutes per week) and categorically (i.e. meeting the national guidelines). The encouraging results obtained through the use of multiple validation approaches may help to address some of the mixed conclusions on the relationship between self-efficacy and PA among Latina women. These mixed conclusions may be a result of methodological differences across studies, as well as the use of different self-efficacy measures.

## Conclusions

In conclusion, this study makes an important contribution to the literature by assessing the psychometrics of the SEPA scale, which to our knowledge have not previously been examined among Latino samples. While the application of behavior theory can be a valuable aspect of health promotion efforts in culturally and ethnically diverse populations, effective use of theory requires a thorough understanding of characteristics of the target population (e.g., ethnicity, socioeconomic status, and gender) [9]. Our study of the widely used SEPA measure contributes to closing the gap in research on the relationship between self-efficacy and objectively measured PA, as well as longer term PA, in Latina women. Accurate measurement of this key theoretical construct can help to understand the relationship between self-efficacy and PA, allowing researchers and health practitioners to more effectively leverage this construct in efforts to reduce the disproportionate burden of lifestyle-related chronic disease in Latina women.

## Abbreviations

7-Day PAR: Seven-Day Physical Activity Recall
IRT: Item Response Theory
MVPA: Moderate to Vigorous Physical Activity
OCC: Option Characteristic Curves
PA: Physical Activity
SCT: Social Cognitive Theory
SEPA: Self-Efficacy for Physical Activity
STOFHLA: Short Test of Functional Health Literacy in Adults
TTM: Transtheoretical Model
